# Reproducibility and Characterization of Head Kinematics During a Large Animal Acceleration Model of Traumatic Brain Injury

**DOI:** 10.3389/fneur.2021.658461

**Published:** 2021-06-09

**Authors:** Andrew R. Mayer, Josef M. Ling, Andrew B. Dodd, Julie G. Rannou-Latella, David D. Stephenson, Rebecca J. Dodd, Carissa J. Mehos, Declan A. Patton, D. Kacy Cullen, Victoria E. Johnson, Sharvani Pabbathi Reddy, Cidney R. Robertson-Benta, Andrew P. Gigliotti, Timothy B. Meier, Meghan S. Vermillion, Douglas H. Smith, Rachel Kinsler

**Affiliations:** ^1^The Mind Research Network/Lovelace Biomedical Research Institute, Albuquerque, NM, United States; ^2^Neurology Department, University of New Mexico School of Medicine, Albuquerque, NM, United States; ^3^Psychiatry Department, University of New Mexico School of Medicine, Albuquerque, NM, United States; ^4^Psychology Department, University of New Mexico School of Medicine, Albuquerque, NM, United States; ^5^Neurosciences Department, University of New Mexico School of Medicine, Albuquerque, NM, United States; ^6^Center for Injury Research and Prevention, Children's Hospital of Philadelphia, Philadelphia, PA, United States; ^7^Department of Neurosurgery and Penn Center for Brain Injury and Repair, Perelman School of Medicine, University of Pennsylvania, Philadelphia, PA, United States; ^8^Department of Neurosurgery, Medical College of Wisconsin, Milwaukee, WI, United States; ^9^Department of Cell Biology, Neurobiology and Anatomy, Medical College of Wisconsin, Milwaukee, WI, United States; ^10^Department of Biomedical Engineering, Medical College of Wisconsin, Milwaukee, WI, United States; ^11^Enroute Care Group, U.S. Army Aeromedical Research Laboratory, Fort Rucker, AL, United States

**Keywords:** traumatic brain injury, large animal model, dynamic acceleration, head kinematics, sensors, diffuse axonal injuries

## Abstract

Acceleration parameters have been utilized for the last six decades to investigate pathology in both human and animal models of traumatic brain injury (TBI), design safety equipment, and develop injury thresholds. Previous large animal models have quantified acceleration from impulsive loading forces (i.e., machine/object kinematics) rather than directly measuring head kinematics. No study has evaluated the reproducibility of head kinematics in large animal models. Nine (five males) sexually mature Yucatan swine were exposed to head rotation at a targeted peak angular velocity of 250 rad/s in the coronal plane. The results indicated that the measured peak angular velocity of the skull was 51% of the impulsive load, was experienced over 91% longer duration, and was multi- rather than uni-planar. These findings were replicated in a second experiment with a smaller cohort (*N* = 4). The reproducibility of skull kinematics data was mostly within acceptable ranges based on published industry standards, although the coefficients of variation (8.9% for peak angular velocity or 12.3% for duration) were higher than the impulsive loading parameters produced by the machine (1.1 vs. 2.5%, respectively). Immunohistochemical markers of diffuse axonal injury and blood–brain barrier breach were not associated with variation in either skull or machine kinematics, suggesting that the observed levels of variance in skull kinematics may not be biologically meaningful with the current sample sizes. The findings highlight the reproducibility of a large animal acceleration model of TBI and the importance of direct measurements of skull kinematics to determine the magnitude of angular velocity, refine injury criteria, and determine critical thresholds.

## Introduction

Although heterogeneous in nature, most human traumatic brain injuries (TBI) are caused by the transmission of energy from an external force to the head that subsequently results in rapid acceleration/deceleration of the brain with or without deformation of the skull (1). Head kinematics have therefore been used to predict TBI pathology in both human and animal models, design safety equipment, and assess the risk of brain injury (2–4). However, to our knowledge, there have only been a handful of large animal studies that have used sensors (5–9) and/or high-speed cameras [see Table 1; (8, 10, 11)] to directly measure the magnitude of head kinematics during acceleration models of injury. To date, no studies have evaluated the reproducibility of head kinematics, which, by definition (65, 66), requires the exact same initial injury conditions to be repeated across multiple animals (i.e., methods reproducibility) and/or in separate experiments (i.e., results reproducibility).

**Table 1:** *In vivo* models of non-invasive diffuse axonal injury.

**Model details**	**Species**	**Journal publication**	**Head kinematics**	**Repd**	**Planes of motion**	**Brief summary of use and publication findings**
Impact: restrained upside-down free fall from height	NHP (mature)	Sano et al. (5)	One uni-axial accelerometer affixed to temporal skull	N	Linear along Z-axis only	Animals fitted with a headgear to attenuate the impact from fall survived at greater heights (up to 8 m) than those animals without a headgear (only up to 6 m)
Non-impact: whiplash resulting from rear impact of cart by pneumatic piston	NHP (mature)	Ommaya et al. (10) Ommaya and Hirsch (11)	HSV	N N	S^a^ S^a^	The presence of concussion (determined by eye movements, apnea, bradycardia, *etc*.) after whiplash was associated with the presence of macroscopic evidence of brain damage
Non-impact: restrained in sled driven by pneumatic piston (HYGE)	NHP (mature)	Masuzawa et al. (8)	Two tri-axial accelerometers affixed to mid-sagittal skull (parietal and occipital) plus HSV	N	Linear along X-axis only	This model reliably induced axonal injury and disrupted electroencephalography (EEG) readings. Accompanied by additional, sometimes fatal, spinal column fractures
Impact: restrained upright in chair, head impacted by piston	NHP (mature)	Ommaya et al. (9) Kanda et al. (7)	Uni-axial linear accelerometer affixed to skull plus HSV Mid-sagittal 9 accelero-meter array plus HSV	N N	S^a^ S	An impact “dose” sufficient to cause concussion occurred at roughly 100 G linear acceleration. Correlation between concussion severity (presence of apnea, diminished pulse, and/or corneal reflex loss) with both EEG amplitude changes and acceleration
Non-impact: head secured in helmet, accelerated by pneumatic actuator (Penn-II)	NHP (age not specified)	Gennarelli et al. (12) Gennarelli and Thibault (13)	Uni-axial accelero-meter rigidly attached to helmet aligned with rotation	N N	C, S, or C/S mixed	Axonal damage produced by pure coronal head acceleration was a major cause of prolonged traumatic coma relative to other methods
Impact: modified captive bolt stunner to unrestrained head	Sheep (toddler)	van den Heuvel et al. (14)	NC	N	A^a^	DAI observable in both the left (impact side) and right brain hemispheres as well as the cerebellum and brain stem 2 h after injury
	Sheep (mature)	Lewis et al. (15) van den Heuvel et al. (16) van den Heuvel et al. (17) van den Heuvel et al. (18) Anderson et al. (6) van den Heuvel et al. (19) Vink et al. (20) Byard et al. (21) Byard et al. (22)	Only Anderson et al. (6) measured head kinematics, with mid-sagittal 9 accelero-meter array and an additional independent reference accelero-meter plus HSV	N N N N N N N N N	A^a^ A^a^ A^a^ A^a^ A^a^ A^a^ A^a^ A^a^ A^a^	The extent of DAI around cerebral contusion in cerebral white matter, central gray matter, cerebellum, and brain stem related to peak change in angular velocity and an index of physiological response to injury. Increased intracranial pressure and decreased cerebral oxygenation were observed after injury, with stabilization or improvement starting 1 h post-injury
	NHP (mature)	Faas and Ommaya (23) Grubb et al. (24) Ommaya et al. (25) Ommaya et al. (26)	HSV in Ommaya et al. (25) and (26) only	N N N N	S^a^ S^a^ S^a^ S^a^	The injury resulted in a decrease in white matter chloride ions. No respiratory abnormalities were observed under normal conditions after injury. Contrecoup contusions were more common for occipital, relative to frontal, impact
	Swine (juvenile)	Finnie et al. (27)	NC	N	A^a^	The use of this model in swine produced substantially less DAI than in sheep at comparable forces.
Non-impact: snout clamped to linkage assembly driven by cyclical motor (*i.e*., shaken baby syndrome model)	Swine (infant)	Coats et al. (28)	NC	N	S or A	Modest DAI produced by repetitive back-and-forth head rotation. DAI increased significantly with time post-injury and had greater red cell neuronal change/extra-axial hemorrhage than a single head rotation 24 h post-injury
Non-impact: snout clamped to linkage assembly driven by pneumatic piston (HYGE; model used in the current study)	Swine (infant)	Raghupathi and Margulies (29) Raghupathi et al. (30) Friess et al. (31) Friess et al. (32) Zhou et al. (33) Coats et al. (34) Naim et al. (35) Eucker et al. (36) Friess et al. (37) Sullivan et al. (38) Clevenger et al. (39) Atlan et al. (2)	NC	N N N N N N N N N N N N	A A A A A S or A A C, S, or A A S S C, S, or A	Axial rotations result in consistent DAI in white matter tracts without tissue tears, with subdural/subarachnoid hemorrhage seen in frontal lobes. Better prediction of injury was achieved when accounting for resistance to rotation. Peak angular acceleration correlates to neurobehavioral deficits and extent of DAI. Axial rotation is more likely to result in ocular hemorrhage than coronal or sagittal, whereas sagittal rotation produces the longest duration of unconsciousness, highest incidence of apnea, largest increase in intracranial pressure, and reduction in cerebral blood flow
	Swine (toddler)	Ibrahim et al. (40) Friess et al. (41) Friess et al. (42) Weeks et al. (43) Friess et al. (44) Jaber et al. (45) Kilbaugh et al. (46) Kilbaugh et al. (47)	NC	N N N N N N N N	A A A S S S S S	Roughly 60% higher peak velocity in axial plane was required to produce similar levels of DAI to swine infant model. The sagittal rotations in this model were mainly used to assess the early effects of medication to improve cerebral perfusion pressure or to investigate mitochondrial dysfunction
	Swine (juvenile)	Ross et al. (48) Wofford et al. (49) Wolf et al. (50) Keating et al. (51)	NC	N N N N	C C or S C S	Initial studies examined dysfunction at the cellular level. More recent studies demonstrate increased pathology for repeat relative to single injury
	Swine (mature)	Meaney et al. (52) Kimura et al. (53) Smith et al. (54) Cecil et al. (55) Smith et al. (56) Chen et al. (57) Smith et al. (58) Smith et al. (59) Stein et al. (60) Chen et al. (61) Browne et al. (62) Johnson et al. (63) Johnson et al. (64)	NC; HSV in *ex vivo* component of Meaney et al. (52) only.	N N N N N N N N N N N N N	C C C C C C C C or A A C C or A C C	Early MRI studies of DAI and longitudinal studies of TBI owing to larger brain size and completed brain development. These studies also represent a shift toward coronal rotational injury. Many of these studies also investigated concentrations of neuronal biomarkers, showing increases in, among others, amyloid beta and immunoglobulin-G as a result of rotational injury, indicating the presence of axonal swelling and the disruption of the blood–brain barrier

*DAI, diffuse axonal injury; Repd, examined reproducibility; HSV, high-speed video capture; NC, not collected; NHP, non-human primates; C, coronal; S, sagittal; A, axial*.

*Species maturity categories were determined by age/weight/author report*.

a *Primary plane of motion determined by impact target (i.e., target on temporal skull has mostly axial translation/rotation; target on frontal or occipital skull has mostly sagittal translation/rotation). Classification of “impact” vs. “non-impact” model based primarily on individual papers*.

Preclinical trauma models are generally categorized into contusional (e.g., weight drop, controlled cortical impact, and fluid percussion injury), blast, penetrating, and acceleration models (67–69). Acceleration models are conceptually similar to blast tertiary injury and are generally considered to be the best model for generating diffuse injury and mimicking human trauma (12, 69). Acceleration injuries have traditionally been classified (see Table 1 for a review) into impact (e.g., bolt guns, sled models with impact) vs. non-impact models (e.g., HYGE, sled models without impact) and can be performed with or without protective equipment (1, 67). Although rodent acceleration models have been proposed (70), examination of the effects of linear and rotational accelerative forces is more practical in gyrencephalic animal species with a larger brain mass (11, 71).

For example, it has been estimated that swine models require an approximately 8-fold increase in acceleration to mimic the forces typically experienced by humans (72). The additional advantages of acceleration models include minimal preparation time (15–20 min) due to lack of craniotomy (73). The primary critiques of acceleration models (1, 69) are related to the financial cost of both instrumentation (non-impact models only) and large animal species themselves, as well as the higher incidence of skull fracture (impact models).

Reproducibility is proposed to be a cornerstone of science (65). However, to our knowledge, there have been no large animal acceleration studies (see Table 1) establishing the reproducibility of head kinematics by either directly mounting a sensor to the animal's head or using high-speed video capture. The majority of previous studies have instead quantified kinematics from the machine used to produce the initial biomechanical forces and assumed a direct correspondence to subsequent head kinematics [e.g., (4)]. The five large animal studies to date that directly measured head kinematics with skull-mounted sensors varied the initial injury load conditions with no repeat tests (5–9). Although these seminal studies were critical for establishing injury criteria and relationships with pathology, they do not permit for the establishment of reproducibility. Head kinematics are routinely used to drive finite element models of head impacts in both animal and human trauma scenarios. Finite element analyses indicate that the magnitude of axonal strains correlates with the velocity and direction of head rotation, moment of inertia, age, and brain size during acceleration (2, 4). Therefore, having accurate measurements of the magnitude of head kinematics is a critical boundary condition for finite element analyses.

The current series of experiments therefore quantified the reproducibility of head kinematics in a swine acceleration model using the HYGE device and examined for potential differences between head and machine kinematics. The HYGE device represents one of the original (12, 13) and more widely used acceleration *non-impact* models (see the discussion and Table 1) and has more recently been adapted for neonatal, juvenile, and adult swine (32, 52, 59, 62). A single angular velocity level (250 rad/s) was targeted in the coronal plane to establish method reproducibility for head kinematics (65) as quantified by the coefficient of variation (COV) across animals. Specifically, the COV is a widely utilized statistical construct to quantify dispersion in multiple scientific fields (66). In the current experimental context, COV quantifies the variability of head kinematic parameters across all animals when the identical impulsive load is targeted. Key parameters (peak angular velocity and pulse duration) were also directly compared between skull- and machine-mounted sensors to establish the transfer of biomechanical forces, with additional regressions performed against immunohistochemistry findings to examine potential relationships with pathology. Finally, we also examined result reproducibility (65) by repeating the experiment a second time in an independent cohort of animals.

## Materials and Methods

### General Animal Procedures for Initial (Experiment 1) and Replication (Experiment 2) Experiments

All animal procedures were approved by our local Institutional Animal Care and Use Committee (IACUC) and the USAMRMC ORP Animal Care and Use Review Office (ACURO). The animals in both experiments were part of a larger study to examine the therapeutic effects of synthetic estrogen on a combined model of TBI and hemorrhagic shock. Sexually mature Yucatan swine were fasted but provided with *ad-libitum* access to water for 6–12 h prior to the experimental procedures. The animals were initially sedated with midazolam (0.5 mg/kg IM injection) and pre-medicated with buprenorphine-SR (0.12 mg/kg IM). The animals were then intubated and maintained under general anesthesia (isoflurane: 5% induction, 1–4% for maintenance combined with oxygen) with a propofol bolus (0.8–1.5 mg/kg) as needed.

**Figure 1:**
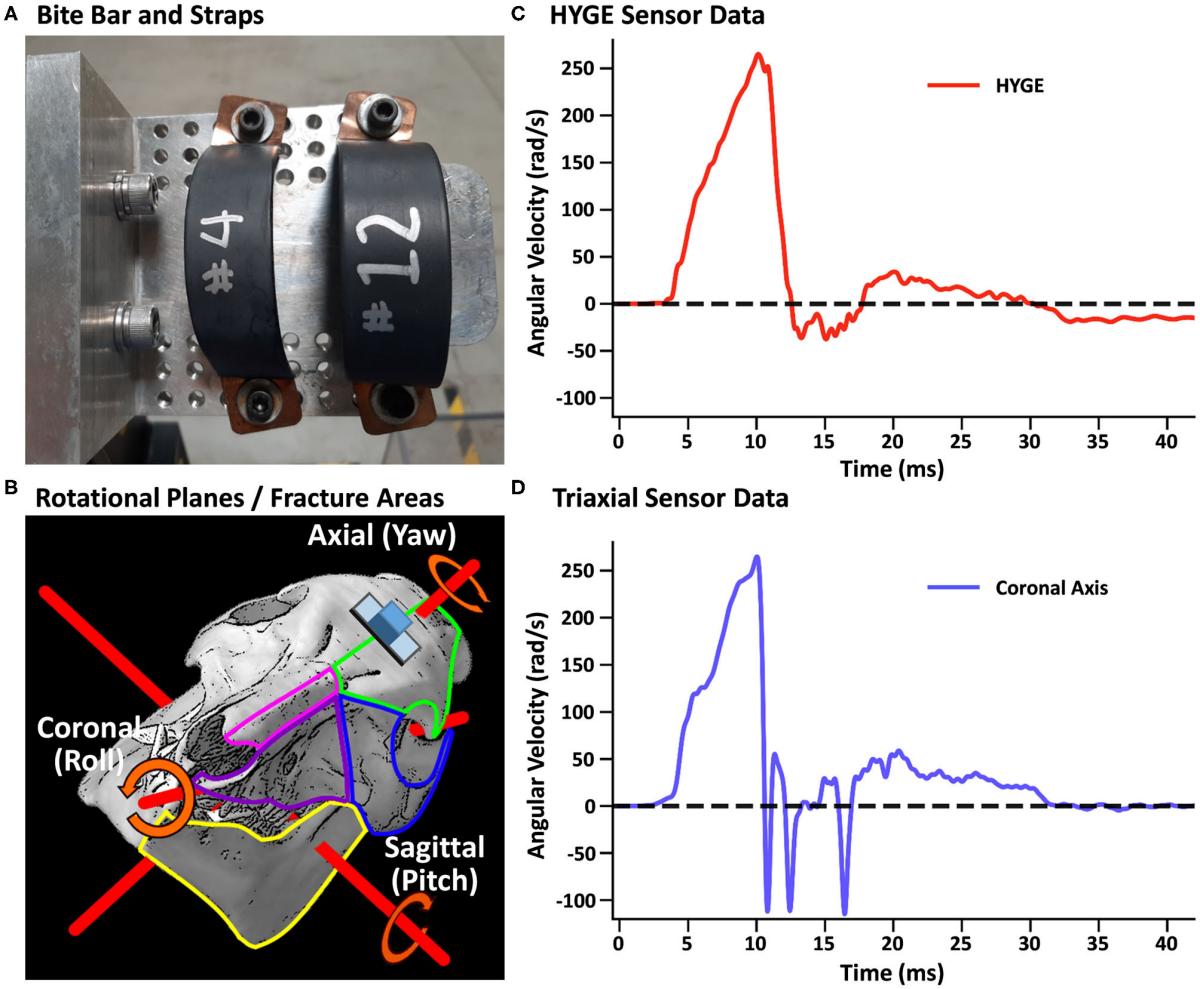
**(A)** Restraint device (bite bar and straps) used in the initial and replication cohorts. **(B)** Representative pig skull depicting the three principal axes (red rods) as well as the placement of the skull sensor (blue cube) and plate (gray rectangle). The skull is rotated 44° along the coronal axis to match the initial starting point of the head when mounted on the restraint device in experiments 1 and 2. The skull also depicts regions of observed fractures (green outline: frontal bone; blue: orbital bone; pink: nasal bone; purple: maxilla; yellow: mandible) recorded in **Table 3**. **(B)** was adapted from University of Texas High-Resolution X-ray CT Facility (NSF IIS-0208675; http://digimorph.org/specimens/Sus_scrofa/skull/). Angular velocity traces (radians/second, rad/s) are shown for the HYGE machine sensor [**(C)**, red trace] and the triaxial sensor [**(D)**, coronal axis = blue trance] when affixed directly onto the bite bar. All angular velocity traces are windowed to include 4 ms of data prior to the identified rise time.

In both experiments, a closed-head rotational TBI was initiated *via* a pneumatic actuator device (HYGE, Inc., Kittanning, PA, USA), similar to a previously described model (73). For the TBI exposure, all animals were maintained under isoflurane (1–4%), with a midazolam IV bolus (0.1–0.5 mg/kg) immediately prior to injury. Isoflurane was disconnected ~30 s prior to the TBI and immediately re-established post-injury. The animals were secured to a custom-made linkage assembly connected to the HYGE device that converts the linear motion of the piston into angular (rotational) motion. The animals' heads were secured (see Supplementary Material) to the linkage assembly through a restraint device comprising a custom-made aluminum alloy bite bar with two straps (Figure 1A). The straps were placed around the snout and bolted into the bite bar. The targeted angular peak velocity for all experiments was 250 rad/s in the coronal plane, with all animals rotated toward their right side.

Machine kinematics were quantified via an in-house data acquisition system using an ARS-06 angular rate sensor (Applied Technologies Associates, Albuquerque, NM, USA; 25 kHz sampling frequency) that was rigidly mounted to the side arm of the HYGE device. A lightweight triaxial angular rate sensor with a small footprint (Diversified Technical Systems ARS3 PRO; 50 kHz sampling rate; 19 × 19 × 12.5 mm, 10 g) was used to directly measure head kinematics during the TBI exposure using a separate data acquisition system than the machine sensor. The sensor was positioned on an aluminum mounting plate whose inferior edge was parallel to a plane extending across the most superior aspects of the orbital sockets, with the plate mid-point located along the longitudinal suture of the skull (see the schematic in Figure 1B and Supplementary Material).

To reduce high-frequency noise, both machine and skull sensor data were smoothed with a four-pole, Butterworth filter (channel frequency class = 1,000 Hz) based on SAE-J211-1 recommendations (74). Spikes in the head sensor data were eliminated from consideration if the maximum was below 100 rad/s, the peak width was <0.4 ms, or the difference between the maximum and neighboring values was >0.3 rad/s. The highest remaining peak was selected as the peak angular velocity. A proxy for impulse duration was calculated by measuring the full width at half-maximum (FWHM) of the peak value (see Supplementary Material). Both peak angular velocity and FWHM from the resultant velocity trace were used as the primary outcome variables to capture any off-axis rotation (i.e., plane of skull near orbital sockets is sloped downward). Time-to-peak (start time defined as 5% of peak velocity) of the resultant served as a secondary outcome variable. The COV (standard deviation divided by the mean) quantified reproducibility at the methods level as has been done in previous biomedical research (66), with COV <5% defined as good, 5–10% as acceptable, and 11–20% as marginal (75, 76).

### Tissue Handling and Neuropathological Evaluation

All animals underwent necropsy, including recording of gross neuropathological findings ~5 h post-injury, as well as immunohistochemistry [Supplementary Material; (77)]. Histopathology was performed on 8-μm-thick sections, focusing at the level of the head of the caudate nucleus and at the vermis of the cerebellum. Single immunohistochemistry labeling was performed to examine for extravasated serum proteins (immunoglobulin G; IgG) as markers of blood–brain barrier integrity and axonal pathology (amyloid precursor protein; APP). All sections were visualized with 3,3′-diaminobenzidine (DAB), counterstained with hematoxylin, dehydrated, and coverslipped. Both negative (no primary antibody) and positive controls were included in each experiment. The immunostained sections were imaged using an Olympus IX71 microscope.

A semi-automated process for APP N-terminus label quantification was carried out using a macro code to allow for simultaneous batch processing of all images by two independent raters separately for each hemisphere. First, the Color Deconvolution plugin in ImageJ Fiji (NIH, Bethesda, MD) was used to separate the hematoxylin stain from the DAB stain by converting the original RGB image into three eight-bit images based on the vector colors for each stain (78). The resulting DAB image was then converted to grayscale and color-inverted. A threshold, determined by calculating the intensity of the lightest APP-positive axonal bulb, was applied to the image to remove background staining and to improve interrater reliability. APP-positive counts were determined using either the original RGB image or a combination of the original RGB (examined for cellular morphology) cross-referenced with the binarized image. The ImageJ Fiji cell counter plugin was utilized to track the count per unit area (877 × 660 μm).

For IgG quantification, six images were taken adjacent to the sulcal depths at the level of the caudate nucleus in addition to six images within the cerebellum. These areas were selected based on vulnerable regions highlighted in previous literature (64) and by differences observed between positive and negative controls in pilot data (77). IgG extravasation was quantified by performing color deconvolution to separate the DAB stain from the hematoxylin stain, calculating the percentage of pixels over a consistent pre-determined background threshold and then averaging the images.

## Results

### Initial Cohort (Experiment 1)

Head and machine kinematic data were successfully collected in nine of 10 attempts (178.3 ± 5.5 days old; 26.4 ± 1.4 kg; four females) during experiment 1, with the mounting plate screws failing during one procedure (bone stripping during placement). Figure 1C depicts the rotational velocity of the sensor mounted directly to the HYGE side arms, while Figure 1D depicts the rotational velocity in the coronal plane of the triaxial skull sensor when mounted directly on the bite bar (i.e., no animal). The results indicate comparable data recorded from both sensors for the coronal axis.

**Figure 2:**
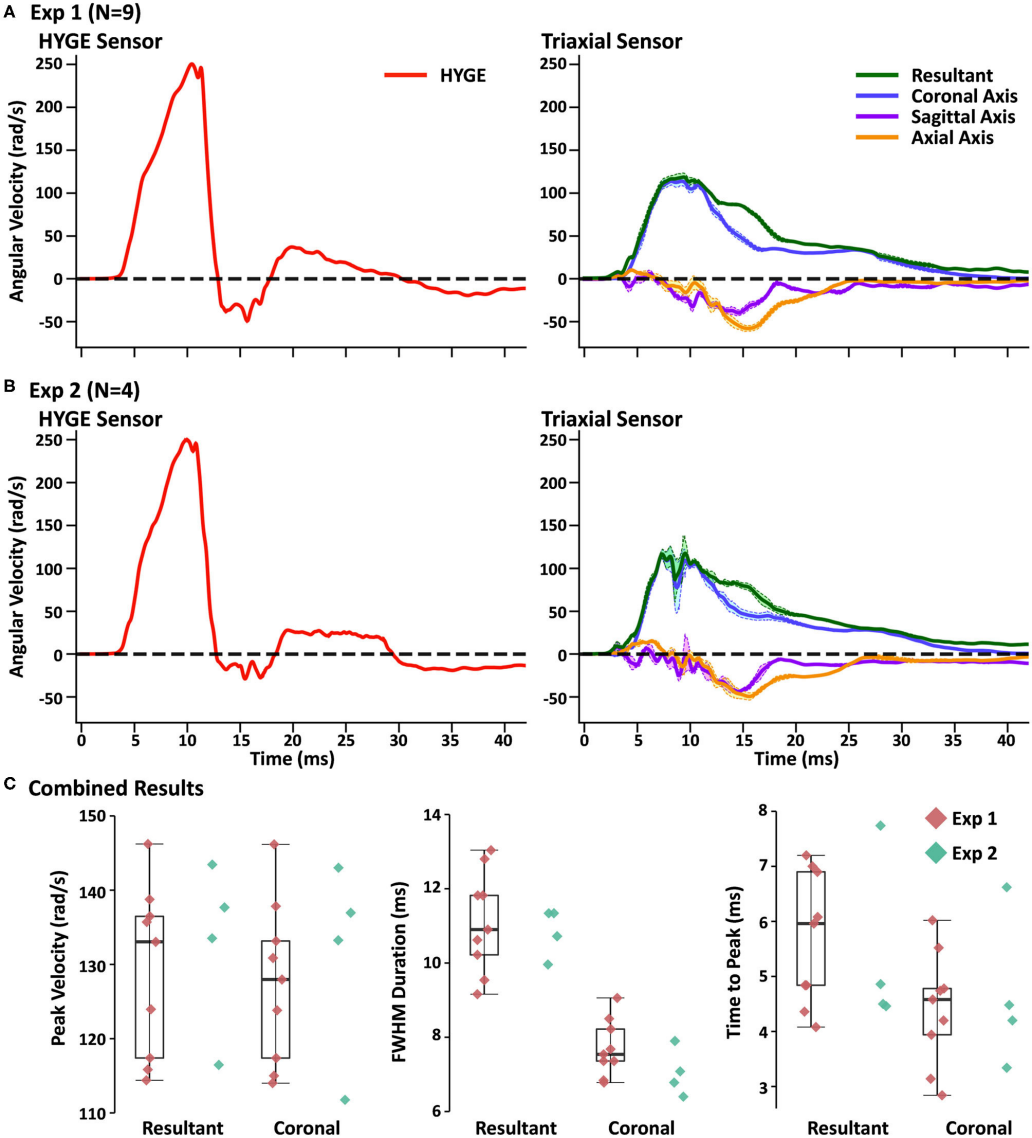
**(A)** Average angular velocity traces (radians per second: rad/s) for experiment (Exp) 1 collected with the HYGE machine sensor (left column; red trace) and triaxial skull sensor (right column; resultant: green trace; coronal axis: blue trace; sagittal axis: purple trace; axial axis: orange trace) in the initial testing cohort (*N* = 9). Off-color shaded bands represent the standard error of the mean for each trace. All angular velocity traces are windowed to include 4 ms of data prior to the identified rise time. **(B)** Plotted data for replication cohort (Exp 2; *N* = 4) machine and skull sensors using an identical scheme. **(C)** Box and scatter plots (Exp 1; red diamonds) or scatter plots (Exp 2; teal diamonds) for peak velocity, full width at half-maximum of the impulse based on peak, and time to peak. Data are plotted separately for the resultant and the coronal axis of the skull sensor data.

Figure 2A and Table 2 depict sensor recordings from the HYGE side arm, the resultant (i.e., combination of all axes) from the triaxial skull sensor, and the angular velocity trace corresponding to the coronal plane. The peak angular velocity of the resultant for the triaxial skull sensor was ~51% (*t*_8_ = 29.00, *p* ≤ 0.001, Morris and Deshon *d* = 15.44) of the magnitude of the HYGE sensor (Figure 2A), with an 8-fold increase in COV (Table 2). The latter was a function of both reduced mean and higher standard deviation (Table 2). A metric of impulse duration (FWHM) from the skull sensor data was significantly (*t*_8_ = −12.06, *p* ≤ 0.001, *d* = −4.39) greater (~91%) than the mean value calculated from the HYGE sensor data. The mean time-to-peak was not statistically different (*p* = 0.09) between the HYGE and the skull sensor data. The comparisons between HYGE and skull sensor recordings in the plane approximating the coronal axis are presented in Supplementary Material.

**Table 2:** Key parameter results for initial testing (experiment 1) and replication (experiment 2) cohorts.

	**HYGE**		**Head: resultant**		**Head: coronal axis**	
	**M ± SD**	**COV%**	**M ± SD**	**COV%**	**M ± SD**	**COV%**
**Experiment 1 (*****N*** **=** **9)**						
Peak angular velocity (rad/s)	250.68 ± 2.88	1.1%	129.09 ± 11.49	8.9%	127.36 ± 10.91	8.6%
FWHM (ms)	5.82 ± 0.15	2.5%	11.1 ± 1.37	12.3%	7.7 ± 0.76	9.8%
Time to peak (ms)	6.45 ± 0.05	0.8%	5.7 ± 1.2	21%	4.42 ± 1.03	23.3%
**Experiment 2 (*****N*** **=** **4)**						
Peak angular velocity (rad/s)	250.13 ± 5.06	2.0%	132.78 ± 11.61	8.7%	131.25 ± 13.60	10.4%
FWHM (ms)	5.82 ± 0.08	1.3%	10.84 ± 0.66	6.0%	7.04 ± 0.64	9.1%
Time to peak (ms)	5.97 ± 0.11	1.9%	5.39 ± 1.58	29.3%	4.66 ± 1.39	29.9%

*M, mean; SD, standard deviation; COV, coefficient of variation; ms, milliseconds; rad/s, radians per second; FWHM, full width at half-maximum*.

The next series of analyses compared skull sensor recordings for the resultant motion relative to the plane approximating the coronal axis. The largest difference was observed for FWHM (*t*_8_ = −9.96, *p* ≤ 0.001, *d* = −2.69), with the resultant exhibiting a longer impulse duration, most likely indicative of multiplanar movement. This can be further confirmed through an examination of the right column of Figure 2A. Consistent multiplanar motion is present in both axes, corresponding with the absolute magnitude of rotational velocity in the sagittal (54.7 ± 14.5 rad/s) and axial (62.6 ± 11.2 rad/s) planes. This multiplanar motion occurred following the peak angular velocity in the coronal plane (see Supplementary Figure 1A for individual sensor data on three randomly selected animals from experiment 1, which further confirms the relatively consistent pattern of head kinematics). Supplementary Video 1 further illustrates the average total angular excursion over time in the three rotation planes.

The resultant motion was also associated with a small but significant (*t*_8_ = −2.48, *p* = 0.03, *d* = −0.15) increase in angular velocity relative to the coronal axis. The time to peak was also significantly higher (*t*_8_ = −6.00, *p* ≤ 0.001, *d* = −1.11) for the resultant relative to coronal plane, although the COVs were roughly similar. Both of these results are expected due to the algorithmic calculation of the resultant (i.e., root mean square summing).

Gross necropsy indicated that all animals in experiment 1 exhibited maxillofacial fractures (herein defined as nasal, frontal, orbit, and mandible bones in the swine) of varying complexity (Table 3). Two animals had fractures that were limited to the left nasal bones only, whereas the other seven animals had more complex fractures that included the left nasal, frontal, and orbit bones. In addition, a right mandible fracture was observed in six animals. Six animals exhibited hemorrhage on the ventral surface of the brain, with one animal also demonstrating hemorrhage that extended from the frontal to the parietal lobes along the longitudinal fissure.

**Table 3:** Fracture distributions for the initial (experiment 1) cohort.

**Fracture area**	**Figure 1B color**	**Left**	**Right**
Frontal bone	Green	7/9	0/9
Orbital bone	Blue	7/9	0/9
Nasal bone	Pink	9/9	0/9
Maxilla	Purple	0/9	0/9
Mandible	Yellow	0/9	6/9

### Replication Cohort (Experiment 2)

Four Yucatan (185.3 ± 10.5 days old; 25.5 ± 1.3 kg) male swine comprised the replication cohort. Figure 2B and Table 2 depict sensor recordings from the HYGE side arm and skull sensor data. All primary results from experiment 1 were replicated, even with the smaller sample size. Specifically, a 46.9% reduction in peak angular velocity magnitude (*t*_3_ = −17.58, *p* ≤ 0.001, *d* = −13.35; Figure 2B) and a significantly increased (86.2%) FWHM (*t*_3_ = 16.84, *p* ≤ 0.001, *d* = 5.37) for the triaxial skull sensor resultant were observed relative to the HYGE sensor (Table 2). Similar to previous findings, the COV was also higher for the skull sensor compared to the HYGE sensor (Table 2). Mean time-to-peak was not statistically different between the HYGE and skull sensor data, with a moderate effect size (*p* = 0.72; *r* = −0.13).

Uncorrected one-vs.-many *t*-tests confirmed that the peak angular velocity (*p* range = 0.08–0.23), FWHM (*p* range = 0.12–0.35), and time to peak (*p* range = 0.06–0.14) for each animal in experiment 2 were statistically similar to those in experiment 1 (Figure 2C; i.e., reproducibility at the single-subject level). The one-vs.-many *t*-tests were not corrected to provide a more liberal threshold for the determination of replication for non-significance. The FWHM for the resultant was also significantly larger than the coronal plane axis (*t*_3_ = 13.19, *p* = 0.001, *d* = 5.88), suggestive of a longer impulse duration (54.3% increase), potentially secondary to multiplanar movement. Specifically, a pattern of consistent magnitudes for the angular velocity components was again present in sagittal (56.3 ± 16.6 rad/s) and axial (54.2 ± 4.4 rad/s) planes (right column of Figure 2B), which temporally corresponded with the peak angular velocity in the coronal plane (see Supplementary Figure 1B for individual experiment 2 animal data).

Similar to experiment 1, gross necropsy indicated that all animals exhibited maxillofacial fractures of varying complexity, which were limited to the left side in all but one animal. Additionally, one animal presented with a right mandible fracture. All four animals exhibited varying degrees of hemorrhage, which was present for the most part on the ventral surface of the brain.

### Necropsy Findings and Correlation With Pathology

**Figure 3:**
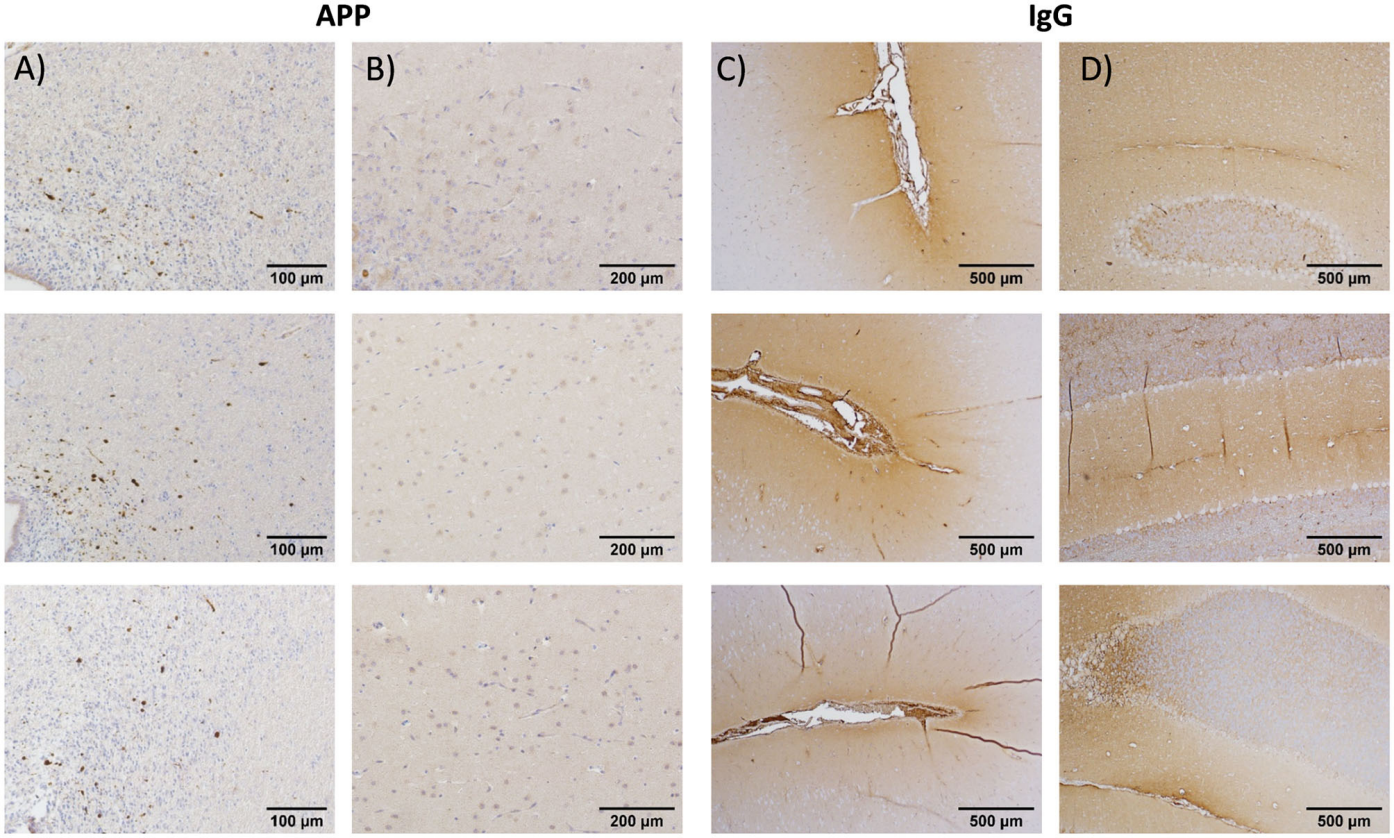
Immunohistochemistry results from, respectively, the periventricular region at the level of the caudate nucleus **(A)** and at the vermis of the cerebellum for amyloid precursor protein **(B)** for selected animals from the initial testing cohort (experiment 1). Periventricular region **(C)** and vermis of cerebellum **(D)**: present data from immunoglobulin G (IgG) antibodies. The results indicated robust evidence of blood–brain barrier breach (IgG) in both the periventricular region and cerebellum, whereas diffuse axonal injury was limited to the cortical and periventricular regions at ~5 h post-injury.

Please see Supplementary Material for interrater reliability results and methods comparisons for experiment 1. Multifocal axonal pathology, characterized by accumulations of APP-positive axonal beads or varicosities, was observed throughout the periventricular section at 5 h post-injury, with the largest accumulation of pathology surrounding the dorsolateral tip of the lateral ventricles (Figure 3A). In contrast, there was minimal evidence of diffuse axonal injury in the vermis of the cerebellum (Figure 3B). IgG extravasation was observed in the depth of the sulci for 9/13 animals and in the vermis of the cerebellum for 11/13 animals (Figures 3C,D).

Given the strong evidence for replication, data were combined across experiments 1 and 2 to determine the potential biological relevance of kinematics in relation to immunohistochemistry findings. Specifically, independent multiple-regression models were used to determine whether the primary measures of angular velocity (peak magnitude and FWHM) from either the HYGE (model 1) or resultant from the skull sensor (model 2) were associated with immunohistochemical evidence of diffuse axonal injury (APP; cross-hemisphere) or blood–brain barrier disruption (IgG; periventricular and cerebellum) across all animals in experiments 1 and 2 (*N* = 13). However, the omnibus tests for all six models were null (*p*'s ≥ 0.21). The correlation coefficients between head kinematics and IHC findings demonstrated medium effect sizes (APP Pearson's *r* = 0.41; IgG *r* = 0.30), suggesting that larger sample sizes would be necessary to detect significant differences in cellular pathology.

### Relationship Between Machine and Skull Sensors

Finally, correlation analyses examined the relationship between the skull sensor resultant motion and HYGE sensor data combined across experiments 1 and 2. There was minimal correlation between the HYGE and skull sensor measurements for peak angular velocity (Pearson's *r* = −0.23; *p* = 0.45), FWHM (*r* = 0.44; *p* = 0.14), or time to peak (*r* = 0.23; *p* = 0.45).

## Discussion

Rapid acceleration/deceleration of the head is potentially the most common factor in human brain trauma (1, 68). However, to date, only five other acceleration studies have mounted sensors directly to the head to quantify potential differences between machine and head kinematics (5–9), and no studies have examined reproducibility. Sano et al. (5) used a gravitational model (free fall from various heights) to induce injury in helmeted and unhelmeted primates, reporting peak linear accelerations of 28–230 g. Masuzawa et al. (8) reported linear accelerations of the head, ranging from 349 to 591 g, and verified with high-speed cameras that there was minimal rotational movement (angular velocity not measured). Ommaya et al. (9) and Kanda et al. (7) used a linear impactor to strike the head of an unrestrained primate, reporting linear head accelerations of up to 869 g (Ommaya) and 830 g (Kanda) and angular velocities of 52–510 rad/s (Kanda). In contrast, the sheep model of Anderson et al. (6) resulted in higher linear accelerations (714–1,835 g) but relatively lower angular velocities (39–118 rad/s), which were attributed to the use of a modified captive bolt gun with higher velocities than the linear impactor of Kanda et al. (7).

The reproducibility of head kinematics has not been examined at either the methodological (individual subjects) or results (across experiments 1 and 2) level for any large animal acceleration model (65). Current results indicate “good” method reproducibility for peak angular velocity (COV = 1.1%) and duration (FWHM COV = 2.5%) for machine kinematics, with exceptional targeting of the desired peak velocity (250.68 ± 2.88 rad/s). However, the mean angular velocity for head kinematics was approximately half of the machine and occurred over a duration that was approximately twice as great. The method reproducibility for these key head kinematic parameters ranged between 8 and 12%, with higher COVs observed for the secondary time-to-peak variable. Critically, the COV produced from the head kinematics data can still be classified in the acceptable or low marginal range based on previous biomechanical models and other biomedical fields (75, 76). There was no statistical relationship between the magnitude of the machine and skull sensor recordings when data were collapsed across experiments, which was likely a result of the extremely high reproducibility from the machine sensor and thus limited variance.

Most dynamic non-impact acceleration models assume that biomechanical forces are limited to a single plane (73), with the plane of rotation influencing clinical (i.e., loss of consciousness) and pathological (i.e., number of hematomas) outcomes in a species-dependent fashion (12, 62). Head kinematics, in the current study, exhibited a complex but relatively consistent pattern of multiplanar motion across both initial and replication cohorts. Specifically, more complex, multiplanar head motion occurred at angular velocities of 50–65 rad/s in both the axial and sagittal planes following the rapid deceleration of the head in the coronal plane. The multiplanar motion was primarily observed as the magnitude of angular velocity declined in the coronal plane, suggesting that it occurred after the snout was fully loaded against the straps. Previous HYGE studies have typically targeted head angular velocities approximately double those observed in the current experiment when principally rotating in the axial (e.g., 142–171 rad/s) or sagittal (e.g., 80–159 rad/s) plane [Table 1; (2, 39, 49, 51)]. However, these previous measurements were obtained from machine-affixed rather than skull sensors. It is therefore difficult to ascertain the biological impact of the multiplanar motion observed in the current study.

Factors related to animal morphometry, biomechanical properties, animal positioning, and/or construction of the restraint device potentially contributed to the observed differences between machine and head kinematics (magnitude and COV). The ratio of head-to-body mass differs among large animals as a function of age (2), species (sheep vs. swine), and strain [Yorkshire vs. Yucatan; (79)], with age and brain mass interacting in a complex fashion to produce axonal injury (2). The width and the length of the snout also likely affect the transfer of energy between the restraint device and the head. Genetically controlling for morphological differences (e.g., snout or head size) is not likely to be economically feasible in large animal models, with genetic modifications also resulting in other unintended consequences observed in other species (80).

Immunohistochemical evidence of diffuse axonal injury (periventricular region only) and blood–brain barrier breach (both periventricular and cerebellar regions) was observed in the initial cohort, replicating previous results observed with this hypovolemic polytrauma injury model (77) as well as multiple previous acceleration models (see Table 1). These two pathologies have been co-localized in other swine rapid acceleration studies using immunoenzymatic double-labeling techniques (64), and the breakdown of the blood–brain barrier (81) may further stimulate the cleavage of APP to release toxic species of Aβ (82). Previous studies have reported associations between immunohistochemically measured diffuse axonal injury and head kinematics (2, 6). However, these studies intentionally varied head kinematics either experimentally (6) or through retrospective data analyses (2), purposefully employing a much larger range of putative inertial load (i.e., measured at the machine level). Additional differences in sample size and statistical power (*N* = 49 in Atlan [2] vs. *N* = 13 in the current study) are also present across experiments. In contrast, a primary focus of experiments 1 and 2 was to examine reproducibility and thus minimize injury variation (e.g., a targeted machine exposure of 250 rad/s in coronal plane for all animals). With these caveats in mind, the current results indicate that the variations observed in head kinematics in the current experiment were not of sufficient magnitude to capture any differences in diffuse axonal injury or blood–brain barrier breach as measured through immunohistochemistry.

Historically, non-impact acceleration models have been characterized by whiplash injury caused by non-cranial strikes (10, 11) and sled crashes (8) or by attaching a helmeted head to the HYGE actuator (12, 13). During the current implementation of the HYGE model, all animals from the initial and replication cohorts exhibited a stereotypical maxillofacial fracture pattern that included the left nasal bones, which sometimes extended bilaterally, or into the orbits. A subset of animals also experienced fractures of the right mandible. The coronal rotation always occurred to the right side of the animal, such that the initial impulsive loading forces from the bite bar (see Figure 1) would maximally impact on the left upper palette/snout and right mandible prior to the rotation of the neurocranium, providing a close correspondence with the observed pattern of maxillofacial fractures. Future studies are required to more carefully delineate which individual or combination of factors principally affects the transfer of biomechanical force between the machine and head. Potential candidates include slippage from the device during the initialization of the injury or the dispersing of energy into maxillofacial structures resulting in fractures.

As previously mentioned, a strength and limitation of the current study was that the experimental procedures were carefully controlled. This is a necessary step to establish reproducibility but limits the generalization of results to the specific experimental methods studied herein. Critically, the transfer function for biomechanical forces between HYGE machine and head may vary based on the initial impulsive loading parameters (e.g., 125 vs. 250 rad/s) and primary plane of rotation (e.g., coronal vs. axial). The financial cost of a series of these experiments would be very high, representing a limitation of any large animal model. Several other limitations to the current study should be noted. First, individual differences in skull morphometry restricted the placement of animals in plumb/identical positions within the restraint device as well as for the placement of the sensor. This limitation is partially mitigated by the consistent head kinematics exhibited by all animals in both the initial and replication cohorts on a group and individual level (see the plots in Supplementary Figure 1) as well as our utilization of the resultant to characterize head kinematics. Second, the current placement of the sensor measured kinematics of the skull rather than the brain, with skull–brain kinematics potentially becoming decoupled during complex trauma (2, 83). Third, only male animals were utilized in the replication experiment, precluding the full generalization of the results to both biological sexes.

In summary, initial impulsive loading parameters such as angular velocity are often used in computational models to predict underlying deformations of brain tissue and the subsequent expression of pathology (2, 3, 28, 83–85). Determining the reproducibility of experienced head kinematics in large animal models is therefore critical for future therapeutic trials seeking to realistically model human trauma in animal surrogates (1, 69). Current results indicate that, for a target peak angular velocity of 250 rad/s in the coronal plane, key head kinematic parameters (angular velocity magnitude and duration) significantly differ from the initial loading conditions produced by the HYGE machine. However, the reproducibility of key head kinematics is generally within acceptable ranges (75, 76). Future studies are necessary to determine if similar results are observed for sagittal and axial rotations using the HYGE device at different angular velocities (2) as well as how other experimental factors (animal weight, body positioning, modifications to the restraint device, etc.) affect the transfer of biomechanical forces between the machine and the head.

## Data Availability Statement

The raw data supporting the conclusions of this article will be made available by the authors, without undue reservation.

## Ethics Statement

The animal study was reviewed and approved by the USAMRMC ORP Animal Care and Use Review Office and Lovelace Biomedical Research Institute's local Institutional Animal Care and Use Committee.

## Author Contributions

AM and RK designed and conceptualized study and aided in collection and analysis of the data. JL wrote the software used to interface with data collection sensors and aided in collection. AD, JR-L, and RD collected, curated, and analyzed data. JL, DS, SP, CR-B, and CM curated and analyzed data. AD and DS performed visualization for figures, tables, and video. AG and MV aided in data collection. AM, JL, AD, DS, RD, CM, SP, and CR-B performed the initial draft of the manuscript. DP, DC, VJ, AG, TM, MV, DS, and RK revised the manuscript for intellectual content and, along with all other authors, approved the final version of the manuscript. All authors contributed to the article and approved the submitted version.

## Conflict of Interest

The authors declare that the research was conducted in the absence of any commercial or financial relationships that could be construed as a potential conflict of interest.
