# Supplementary material for: Reproducibility and Characterization of Head Kinematics During a Large Animal Acceleration Model of Traumatic Brain Injury
Source: Front Neurol. 2021 Jun 9;12:658461. doi: 10.3389/fneur.2021.658461 (PMC8219951; doi:10.3389/fneur.2021.658461)
Supplement: Supplementary file 1 [file Data_Sheet_1.docx]

***Supplemental Methods***

All animals were placed in ventral (sternal) recumbency in sphinx position. The spinal column was horizontally aligned with the point of rotation (i.e., center of bite bar mount), with the center of the cervical column positioned on a plane that was slightly above the point of rotation. The posterior strap was positioned to be as spatially proximal to the nasium as possible, with the second strap positioned just anterior to first strap. A range of strap sizes in 1/8 inch length increments were available to maximize strap fit.

A sensor for measuring head kinematics was positioned on an aluminum mounting plate whose inferior edge was parallel to a plane extending across the most superior aspects of the orbital sockets, with the plate mid-point located along the longitudinal suture of the skull (see schematic in Figure 1B). Four skin punches were initially made on the scalp corresponding to the location of the screw holes in the mounting plate template. Pilot holes were then drilled, followed by rigid attachment of the plate to the skull with 14 mm cortical screws. The sensor was then rigidly mounted to the plate.

Resultant angular velocity of the head was calculated by squaring and summing angular velocity components from the three unique axes of rotation. Peak resultant angular velocity and full-width at half maximum (FWHM) for the resultant were used as the primary outcome variables. Full width at half maximum was a proxy measure for duration, and was calculated by estimating the width (unit = milliseconds) of the impulse at the peak duration. FWHM was chosen given the more complex and longer kinematics experienced by animals relative to the machine sensor (see Figure 2A and 2B),with graphical representations presented in Supplemental Figure 2.The mounted sensor experienced off-axis rotation (i.e., plane of skull near orbital sockets is sloped downward), which varied owing to differences in individual animal morphometry. The resultant therefore represents a surrogate of both uniplanar and multiplanar head motion, as well as addressing individual differences in mounting procedures.

All animals in Experiments 1and 2 underwent necropsy(1)5 hours post-injury and had any gross neuropathological findings recorded. Retrograde aortic perfusion was performed with ambient temperature heparinized (10 IU/ml) phosphate-buffered saline, followed by ambient temperature 10% neutral buffered formalin. Following 24 hours of fixation, the brain was removed from the skull and immersed in 10% neutral buffered formalin for 7 days. The brain was immediately trimmed (whole brain coronal blocks at 5mm intervals), processed, and paraffin embedded.

Histopathology was performed on 8-micron thick sections, focusing at the level of the head of the caudate nucleus and at the vermis of the cerebellum. Single immunohistochemistry labeling was performed to examine for extravasated serum proteins (Immunoglobulin G; IgG: Vector Labs, Burlingame, CA; 1:2,000) as markers of blood-brain barrier integrity and axonal pathology (amyloid precursor protein; APP; Millipore, Billerica, MA; 1:90,000). Sections were first deparaffinized in xylene and rehydrated through descending grades of ethanol. Tissues were then quenched for endogenous peroxidase activity with 3% hydrogen peroxide for 15 minutes and heat-induced antigen retrieval was performed using Tris-EDTA buffer (pH 8.0) in a pressure cooker for 8 minutes. Tissues were blocked in 1% normal horse serum for 30 minutes at room temperature and incubated at 4°C overnight with primary antibody recognizing amino acids 66-81 of the APP N-terminus (Millipore, Billerica, MA; 1:90,000) or biotinylated anti-swine IgG (Vector Labs, Burlingame, CA; 1:2,000). After washes with phosphate-buffered saline, sections were incubated with a biotinylated secondary antibody for 30 minutes, washed again in phosphate-buffered saline, and incubated with avidin-biotin complex (Universal Elite kit, Vector Labs, Burlingame, CA) for 30 minutes. Sections were then visualized with 3, 3’-diaminobenzidine (DAB), counterstained with hematoxylin, dehydrated, and coverslipped. A negative control (no primary antibody) was included in each experiment to control for non-specific binding. Immunostained sections were imaged using an Olympus IX71 microscope.

Effects sizes were calculated using the method recommended by Morris and Deshon to account for shared variance (2).

***Results***

*Initial Cohort (Experiment 1)*

When comparing the HYGE sensor to the coronal axis of the head sensor, the result was similar to that observed of the HYGE versus resultant comparison for mean angular velocity (*t*_8_=31.53, *p*≤0.001, *d*=16.03; 49% reduction), in addition to the FWHM comparison (t_8_= -7.15,p≤0.001,d=-3.57), albeit at a lower percentage decrease (25%) of the HYGE relative to coronal axis estimates. Converse to the null finding between HYGE and the resultant, time-to-peak was significantly different(*t*_8_=5.91, *p*≤0.001, *d*=2.93)between the HYGE sensor(6.5±0.0ms; COV= 0.8%) and the coronal axis(4.4±1.0ms; COV= 23.3%). See Table 2 for full descriptive values.

APP N-terminus label quantification demonstrated excellent interrater reliability across both raters for the periventricular region (intraclass correlation coefficient for left hemisphere=0.998; right hemisphere=0.995) for Experiment 1 animals. Intraraterreliability was also excellent (0.993) for APP-positive counts between the RGB (1.33×10^-4^±1.13×10^-4^ parts per area unit) and the RGB plus binarized images (1.44×10^-4^±1.21×10^-4^ parts per area unit), with a higher count present for the RGB plus binarized images due to improved ability to more consistently visualize varicosities on the binarized images with a fixed threshold level (*Wald-χ^2^* = 138.77; *p* < 0.001).

**References**

(1) Mayer AR, Dodd AB, Ling JM, Stephenson DD, Rannou-Latella JG, Vermillion MS, et al. Survival rates and biomarkers in two large animal models of combined traumatic brain injury and hemorrhagic shock: Preliminary Results. Shock 2020.

(2) Morris SB, DeShon RP. Combining effect size estimates in meta-analysis with repeated measures and independent-groups designs. Psychol Methods 2002 Mar;7(1):105-25.


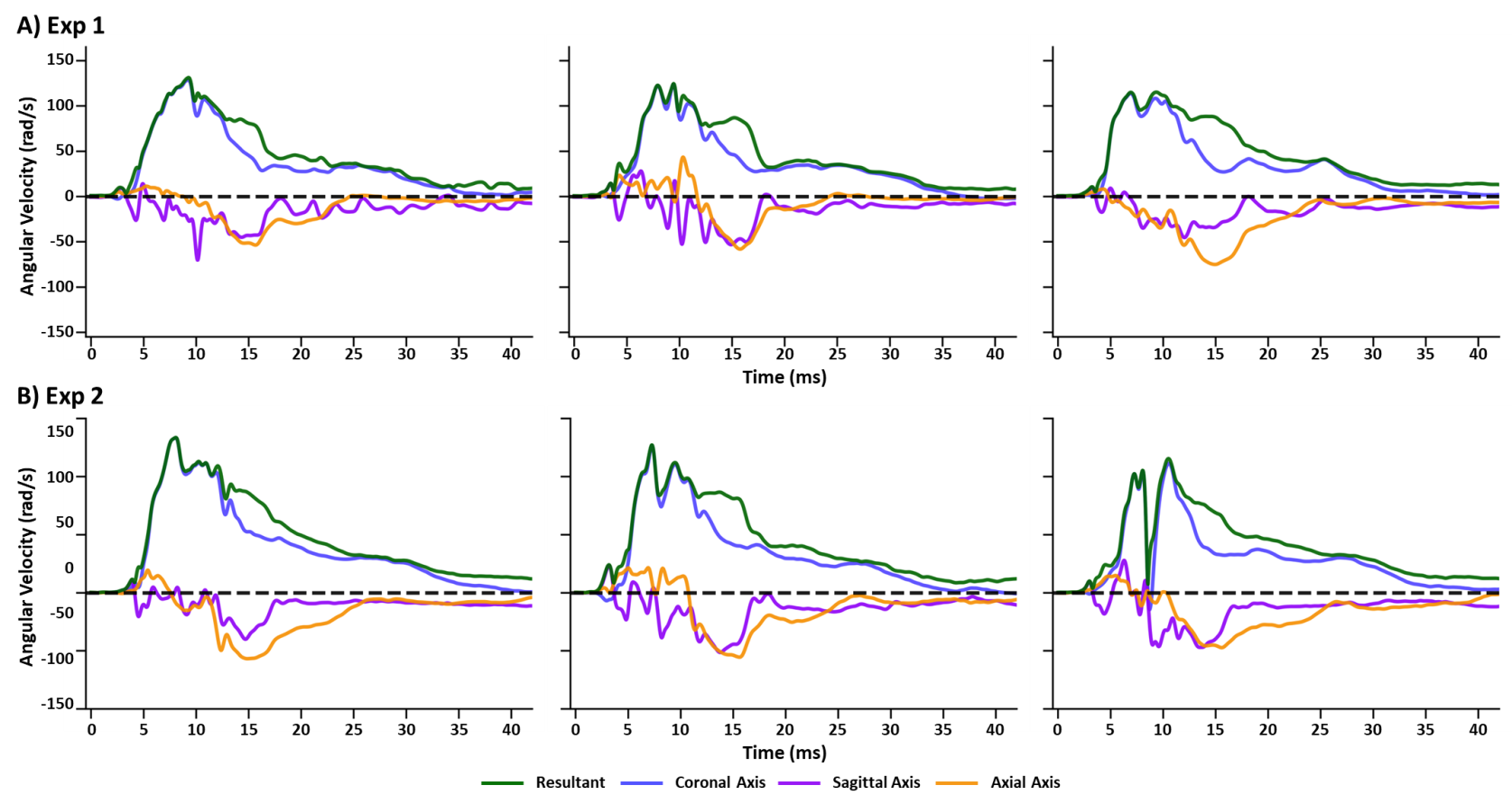


**Supplemental Figure 1:**Panel A presents angular velocity traces (radians per second: rad/s) for three animals randomly selected from the initial cohort (Experiment 1; Exp 1 N=9) collected with the triaxial head sensor for all three axis (coronal = blue trace; sagittal = purple trace; axial = orange trace) as well as the resultant (green trace). Panel B presents angular velocity for three randomly selected animals (N=4) in the replication cohort (Experiment 2; Exp 2). Although slight differences are observed across the individual animals, the pattern of recorded head kinematics are very consistent for coronal, sagittal and axial planes. All angular velocity traces are windowed to include 4 ms of data prior to the identified rise time.


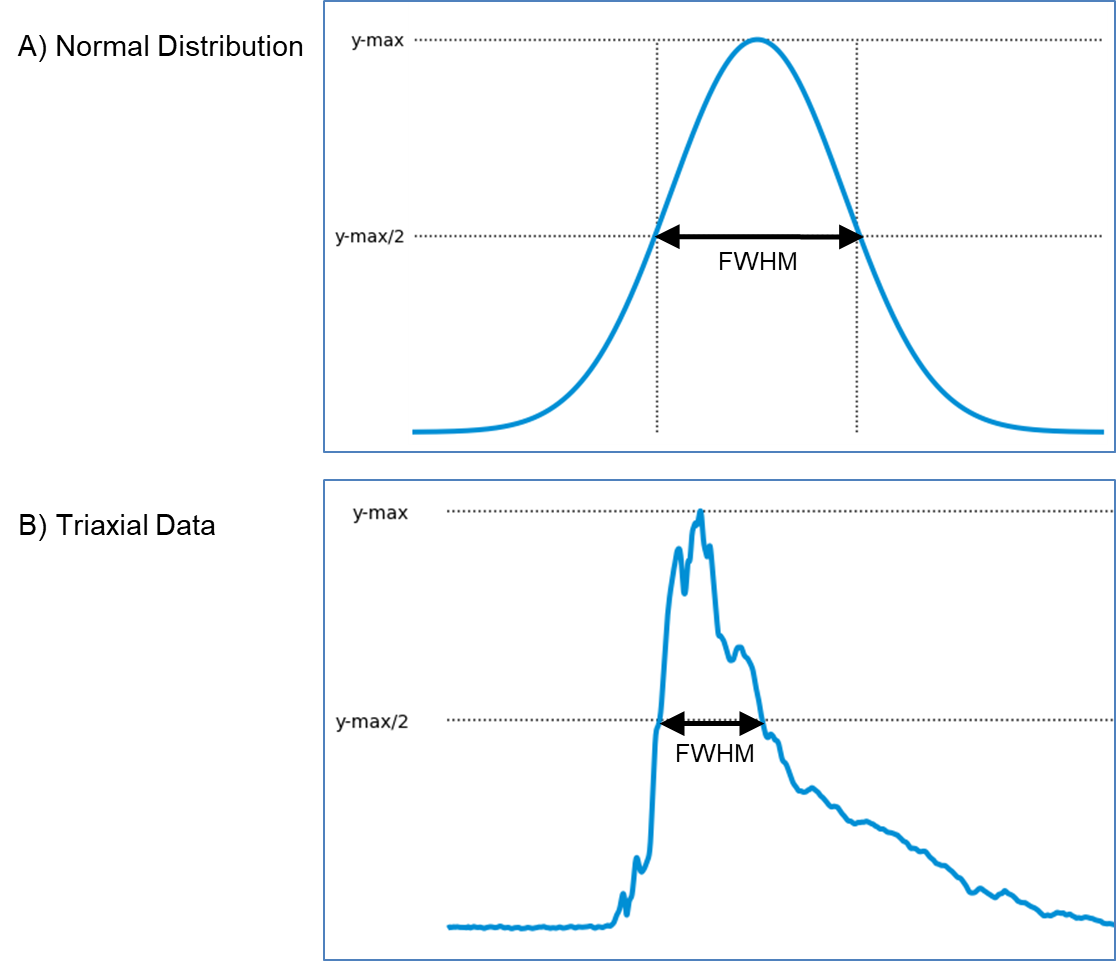


**Supplemental Figure 2:**Panel A shows a diagram of how full-width at half-maximum is identified on a normal distribution. Panel B shows how it is realized for triaxial resultant data.

**Supplemental Video 1:**This video depicts the average of the angular rotation (degrees [deg]) over time experienced by all animals in the initial cohort (Experiment 1) in the coronal (blue), axial (purple), and sagittal (orange) planes. The swine head used in the panels is derived from an MRI collected in a previous study, but is representative of the species and strain used in Experiments 1 and 2. Playback is at 1/200 of normal speed.
